# Applications of the MapReduce programming framework to clinical big data analysis: current landscape and future trends

**DOI:** 10.1186/1756-0381-7-22

**Published:** 2014-10-29

**Authors:** Emad A Mohammed, Behrouz H Far, Christopher Naugler

**Affiliations:** 1 Department of Electrical and Computer Engineering, Schulich School of Engineering, University of Calgary, Calgary, AB, Canada; 2 Department of Pathology and Laboratory Medicine, University of Calgary and Calgary Laboratory Services, Calgary, AB, Canada

**Keywords:** MapReduce, Hadoop, Big data, Clinical big data analysis, Clinical data analysis, Bioinformatics, Distributed programming

## Abstract

The emergence of massive datasets in a clinical setting presents both challenges and opportunities in data storage and analysis. This so called “big data” challenges traditional analytic tools and will increasingly require novel solutions adapted from other fields. Advances in information and communication technology present the most viable solutions to big data analysis in terms of efficiency and scalability. It is vital those big data solutions are multithreaded and that data access approaches be precisely tailored to large volumes of semi-structured/unstructured data.

The MapReduce programming framework uses two tasks common in functional programming: Map and Reduce. MapReduce is a new parallel processing framework and Hadoop is its open-source implementation on a single computing node or on clusters. Compared with existing parallel processing paradigms (e.g. grid computing and graphical processing unit (GPU)), MapReduce and Hadoop have two advantages: 1) fault-tolerant storage resulting in reliable data processing by replicating the computing tasks, and cloning the data chunks on different computing nodes across the computing cluster; 2) high-throughput data processing via a batch processing framework and the Hadoop distributed file system (HDFS). Data are stored in the HDFS and made available to the slave nodes for computation.

In this paper, we review the existing applications of the MapReduce programming framework and its implementation platform Hadoop in clinical big data and related medical health informatics fields. The usage of MapReduce and Hadoop on a distributed system represents a significant advance in clinical big data processing and utilization, and opens up new opportunities in the emerging era of big data analytics. The objective of this paper is to summarize the state-of-the-art efforts in clinical big data analytics and highlight what might be needed to enhance the outcomes of clinical big data analytics tools. This paper is concluded by summarizing the potential usage of the MapReduce programming framework and Hadoop platform to process huge volumes of clinical data in medical health informatics related fields.

## Introduction

Big data is the term used to describe huge datasets having the “4 V” definition: volume, variety, velocity and value (e.g. medical images, electronic medical records (EMR), biometrics data, etc.). Such datasets present problems with storage, analysis, and visualization [1,2]. To deal with these challenges, new software programming frameworks to multithread computing tasks have been developed [2-4]. These programming frameworks are designed to get their parallelism not from a supercomputer, but from computing clusters: large collections of commodity hardware, including conventional processors (computing nodes) connected by Ethernet cables or inexpensive switches. These software programming frameworks begin with a new form of file system, known as a distributed file system (DFS) [3,4], which features much larger units than the disk blocks in a conventional operating system. DFS also provides replication of data or redundancy to protect against the frequent media failures that occur when data is distributed over potentially thousands of low cost computing nodes [3]. The goal of this review is to summarize the potential and expanding usage of MapReduce on top of the Hadoop platform in the processing of clinical big data. A secondary objective is to highlight the potential benefits of predictive and prescriptive clinical big data analytics. These types of analytics are needed for better usage and optimization of resources [5,6].

### Types of analytics

Analytics is a term used to describe various goals and techniques of processing a dataset.

There are three types of analytics:

1- Descriptive analytics: is a process to summarize the dataset under investigation. It may be used to generate standard reports that might be useful to address questions like “What happened? What is the problem? What actions are needed?”

2- Predictive analytics: descriptive analytics, unfortunately do not tell anything about the future, that is the reason predictive analytics is needed. Predictive analytics utilize statistical models of the historical datasets to predict the future. Predictive analytics are useful to answer questions like “Why is this happening? What will happen next?”. The predictive ability is dependent on the goodness of fit of the statistical model [6].

3- Prescriptive analytics: are the type of analytics that help in utilizing different scenarios of the data model (i.e. multi-variables simulation, detecting hidden relationships between different variables). It is useful to answer questions like “What will happen if this scenario of resource utilization is used? What is the best scenario?”. Prescriptive analytics are generally used in optimization problems and require sophisticated algorithms to find the optimum solution and therefore are less widely used in some fields (i.e. clinical big data analytics).

This paper summarizes the efforts in clinical big data analytics which currently entirely focus on descriptive and predictive analytics. This in turn is followed by a discussion of leveraging clinical big data for analytical advantages and highlighting the potential importance of prescriptive analytics with potential applications that might arise from these types of analyses. (See section on Clinical big data and upcoming challenges).

### High Performance Computing (HPC) systems

#### Distributed system

A distributed system [3] is a setup in which several independent computers (computing nodes) participate in solving the problem of processing a large volume of and variety of structured/semi-structured/unstructured data.

#### Grid computing system

The grid computing system [7] is a way to utilize resources (e.g. CPUs, storage of computer systems across a worldwide network, etc.) to function as a flexible, pervasive, and inexpensive accessible pool of computing resources that can be used on demand by any task.

#### Graphical processing unit (GPU)

GPU computing [8] is well adapted to the throughput-oriented workload problems that are characteristic of large-scale data processing. Parallel data processing can be handled by GPU clusters [9]. However, implementing MapReduce on a cluster of GPUs has some limitations [10]. For example GPUs have difficulty communicating over a network. Moreover GPUs cannot handle virtualization of resources. Furthermore the system architecture of GPUs may not be suitable for the MapReduce architecture and may require a great deal of modification [9].

The basic differences between grid computing and distributed computing systems are:

1. A distributed computing system manages hundreds or thousands of computer systems, which are limited in processing resources (e.g. memory, CPU, storage, etc.). However the grid computing system is concerned about efficient usage of heterogeneous systems with optimal workload management servers, networks, storage, etc.

2. A grid computing system is dedicated to support computation across a variety of administrative domains, which makes it different from the traditional distributed computing system.

### Distributed file systems

**Figure 1:**
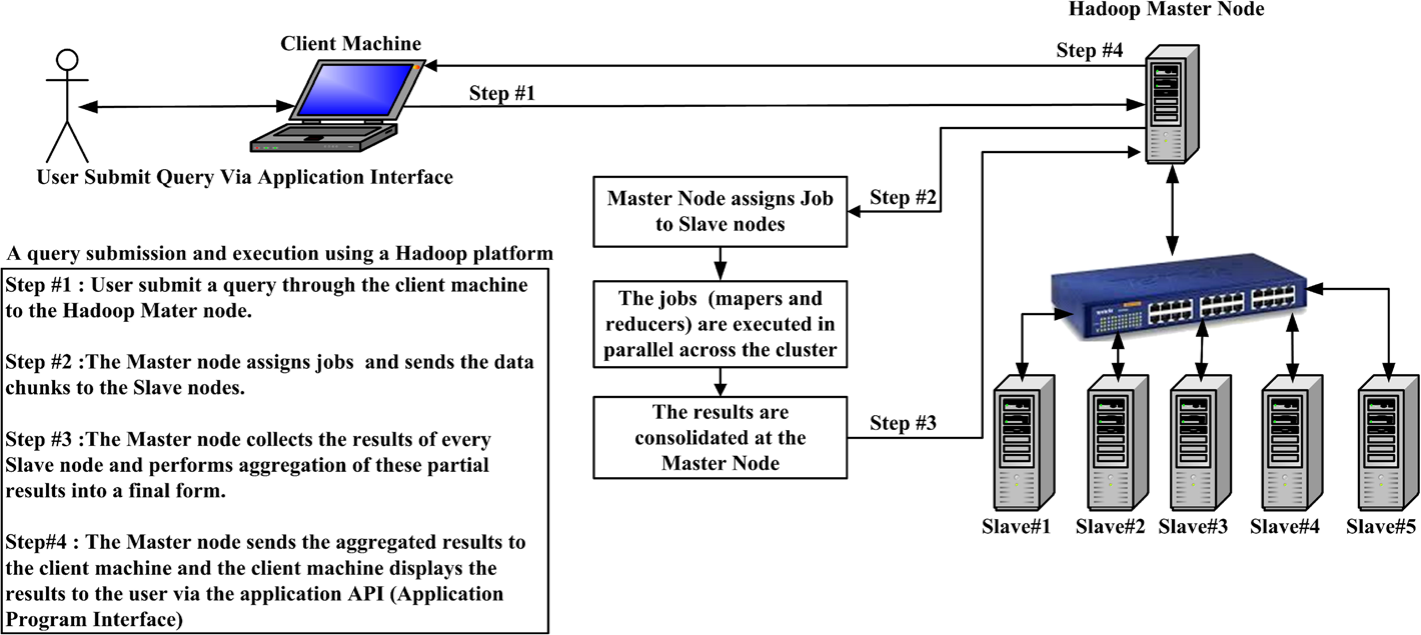
**The architecture of the Hadoop cluster.** Hadoop cluster architecture, showing the distributed computing nodes, which are Master node (NameNode), Slave Nodes (DataNode), and the Ethernet switch.

Most computing is done on a single processor, with its main memory, cache, and local disk (a computing node). In the past, applications that called for parallel processing, such as large scientific calculations, were done on special-purpose parallel computers with many processors and specialized hardware [2,3]. However, the prevalence of large-scale Web services has resulted in more computing being done on installations with thousands of computing nodes operating more or less independently [3,4]. In these installations, the computing nodes are commodity hardware, which greatly reduces the cost compared to special-purpose parallel machines [3]. These new computing facilities have given rise to a new generation of programming frameworks. These frameworks take advantage of the power of parallelism and at the same time avoid the reliability problems that arise when the computing hardware consists of thousands of independent components, any of which could fail at any time [2]. Figure 1 shows a Hadoop cluster with its distributed computing nodes and connecting Ethernet switch. The cluster runs jobs controlled by the master node, which is known as the NameNode and it is responsible for chunking the data, cloning it, sending the data to the distributed computing nodes (DataNodes), monitoring the cluster status, and collecting/aggregating the results. The cluster illustrated in Figure 1 is currently installed in the Department of Pathology and Laboratory Medicine, University of Calgary and Calgary Laboratory Services (CLS), Calgary, Alberta, Canada.

### The MapReduce programming framework

On top of the DFS, many different higher-level programming frameworks have been developed. The most commonly implemented programming framework is the MapReduce framework [4,11,12]. MapReduce is an emerging programming framework for data-intensive applications proposed by Google. MapReduce borrows ideas from functional programming [12], where the programmer defines Map and Reduce tasks to process large sets of distributed data.

Implementations of MapReduce [11] enable many of the most common calculations on large-scale data to be performed on computing clusters efficiently and in a way that is tolerant of hardware failures during computation. However MapReduce is not suitable for online transactions [11,12].

**Figure 2:**
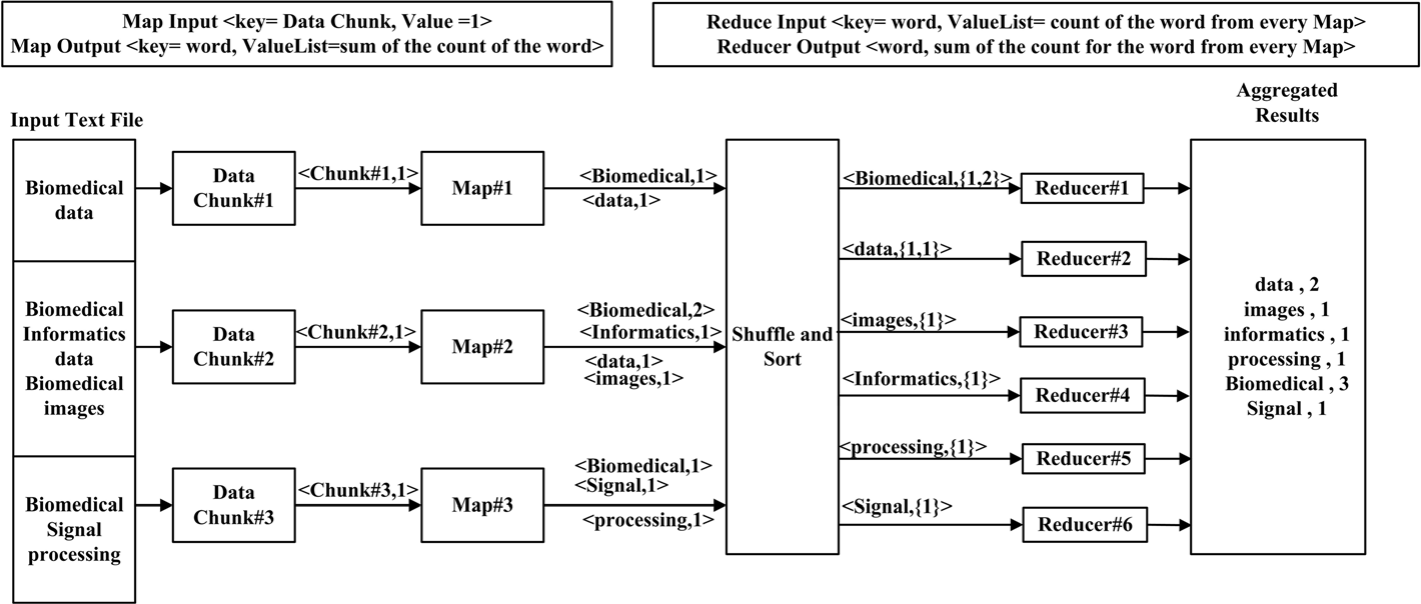
**The WordCount problem MapReduce algorithm workflow.** The algorithm counts the number of occurrences for every word in the file. The file is chunked and distributed over the computing nodes in the cluster. The mapper must be completed to start the reducer phase, otherwise an error will be reported and the execution will be stopped.

The key strengths of the MapReduce programming framework are the high degree of parallelism combined with the simplicity of the programming framework and its applicability to a large variety of application domains [4,11]. This requires dividing the workload across a large number of machines. The degree of parallelism depends on the input data size. The map function processes the input pairs (key1, value1) returning some other intermediary pairs (key2, value2). Then the intermediary pairs are grouped together according to their key. The reduce function will output some new key-value pairs of the form (key3, value3). Figure 2 shows an example of a MapReduce algorithm used to count words in a file. In this example the map input key is the provided data chunk with a value of 1. The map output key is the word itself and the value is 1 every time the word exists in the processed data chunk. The reducers perform the aggregation of the key-values pair output from the maps and output a single value for every key, which in this case is a count for every word. Figure 2 provides further explanation of the generation of the key-value pairs produced during the processing phases of the WordCount MapReduce program.

High performance is achieved by breaking the processing into small units of work that can be run in parallel across potentially hundreds or thousands of nodes in the cluster. Programs written in this functional style are automatically parallelized and executed on a large cluster of commodity machines. This allows programmers without any experience with parallel and distributed systems to easily utilize the resources of a large distributed system [3,4].

MapReduce programs are usually written in Java; however they can also be coded in languages such as C++, Perl, Python, Ruby, R, etc. These programs may process data stored in different file and database systems.

### The hadoop platform

Hadoop [13-15] is an open source software implementation of the MapReduce framework for running applications on large clusters built of commodity hardware from Apache [16]. Hadoop is a platform that provides both distributed storage and computational capabilities. Hadoop was first comprehended to fix a scalability issue that existed in Nutch [15,17], an open source crawler and search engine that utilizes the MapReduce and big-table [17] methods developed by Google. Hadoop is a distributed master–slave architecture that consists of the Hadoop Distributed File System (HDFS) for storage and the MapReduce programming framework for computational capabilities. The HDFS stores data on the computing nodes providing a very high aggregate bandwidth across the cluster.

Traits inherent to Hadoop are data partitioning and parallel computation of large datasets. Its storage and computational capabilities scale with the addition of computing nodes to a Hadoop cluster, and can reach volume sizes in the petabytes on clusters with thousands of nodes.

Hadoop also provides Hive [18,19] and Pig Latin [20], which are high-level languages that generate MapReduce programs. Several vendors offer open source and commercially supported Hadoop distributions; examples include Cloudera [21], DataStax [22], Hortonworks [23] and MapR [24]. Many of these vendors have added their own extensions and modifications to the Hadoop open source platform.

Hadoop differs from other distributed system schemes in its philosophy toward data. A traditional distributed system requires repeat transmissions of data between clients and servers [3]. This works fine for computationally intensive work, but for data-intensive processing, the size of data becomes too large to be moved around easily. Hadoop focuses on moving code to data instead of vice versa [13,14]. The client (NameNode) sends only the MapReduce programs to be executed, and these programs are usually small (often in kilobytes). More importantly, the move-code-to-data philosophy applies within the Hadoop cluster itself. Data is broken up and distributed across the cluster, and as much as possible, computation on a chunk of data takes place on the same machine where that chunk of data resides.

**Figure 3:**
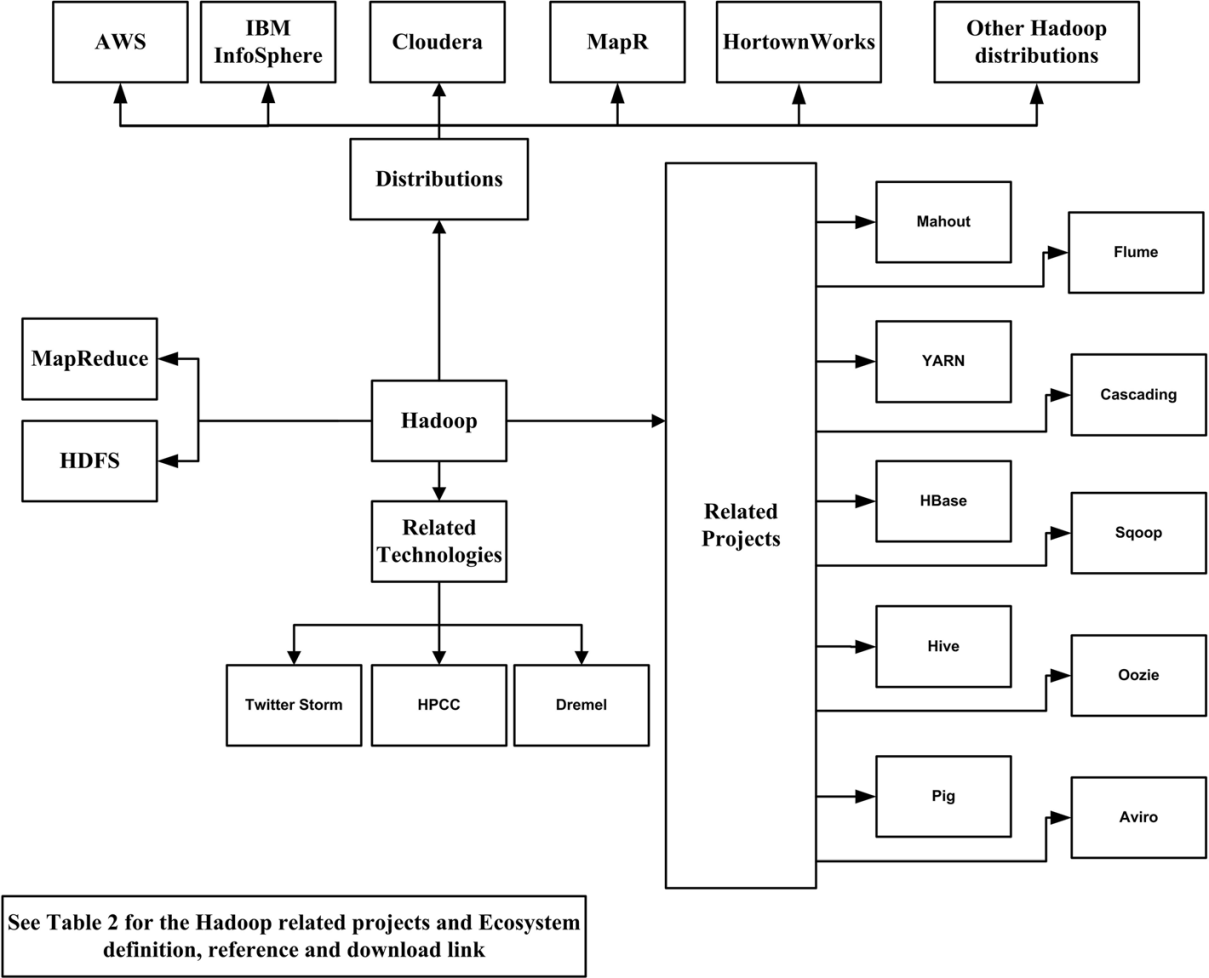
**The Hadoop ecosystems.** The Hadoop system core, components (ecosystems), associated technology, and different distributions by vendors. This Figure illustrates the current Hadoop ecosystem and a short list of the available distributions by vendors.

Figure 3 shows the Hadoop ecosystems, the associated technology, and the current distribution existing in the market. Table 1 shows the basic features of 14 Hadoop distributions [25] and Table 2 shows the related Hadoop projects/ecosystems that are used on top of the Hadoop to provide my functionalities to the MapReduce framework.

**Table 1:** Basic features of 14 Hadoop distributions and related download links

**Vendor**	**Features**	**Download URL**
Amazon Web Services Inc	• Amazon Elastic Block Store	http://aws.amazon.com/
	• Amazon Virtual Private Cloud	
	• GPU Instances	
	• High Performance Computing (HPC) Cluster	
IBM Corp	• Social and Machine Data Analytics Accelerator	http://www-03.ibm.com/software/products/en/infobigienteedit/
	• Provides a workload scheduler	
	• Includes Jaql, a declarative query language.	
	• Allows executing R jobs directly from the BigInsights web console.	
Pivotal Corp	• A Fast, Proven SQL Database Engine for Hadoop	http://www.gopivotal.com/products/pivotal-hd
	• Enterprise Real-Time Data Service on Hadoop	
	• Familiar SQL Interface	
	• Hadoop In the Cloud: Pivotal HD Virtualized by VMware	
Cloudera Inc	• HDFS Snapshots	http://www.cloudera.com/content/cloudera/en/products-and-services/cloudera-enterprise.html
	• Support for running Hadoop on Microsoft Windows	
	• YARN API stabilization	
	• Binary Compatibility for MapReduce applications built on hadoop-1.x	
MapR Technologies Inc	• Finish small jobs quickly with MapR ExpressLane	http://www.mapr.com/products/only-with-mapr
	• Enable atomic, consistent point-in-time recovery with MapR Snapshots	
Hortonworks Inc	• Use rich business intelligence (BI) tools such as Microsoft Excel, PowerPivot for Excel and Power View	http://hortonworks.com/products/hdp/
	• HDP for Windows is the ONLY Hadoop distribution available for Windows Server.	
Karmasphere Inc	• Ability to Use Existing SAS, SPSS and R Analytic Models	http://www.karmasphere.com/product-overview/key-features/
Hadapt Inc	• Analyze both structured and unstructured data in a single, unified platform	http://hadapt.com/product/
Super Micro Computer Inc	• Fully-validated, pre-configured SKUs optimized for Hadoop solutions	http://www.supermicro.com/products/rack/hadoop.cfm
Pentaho Corp	• Visual development for Hadoop data preparation and modeling	http://www.pentahobigdata.com/ecosystem/platforms/hadoop
Zettaset Inc	• Enterprise-Grade Hadoop Cluster Management	http://www.zettaset.com/platform.php
Datastax Inc	• Powered by Apache Cassandra™, Certified for Production	http://www.datastax.com/what-we-offer/products-services/datastax-enterprise/apache-hadoop
Datameer Inc	• Data Integration, Analytics, and Visualization	http://www.datameer.com/
Dell Inc	• Cloudera distribution for Hadoop	http://www.dell.com/learn/us/en/555/solutions/hadoop-big-dataSolution?c=us&l=en&s=biz&cs=555

**Table 2:** Description of the Hadoop related projects/ecosystems

**Hadoop related project and technology**	**Description**	**Download URL**
Avro	• Avro is a framework for performing remote procedure calls and data serialization.	http://avro.apache.org
Flume	• Flume is a tool for harvesting, aggregating and moving large amounts of log data in and out of Hadoop.	http://flume.apache.org
HBase	• Based on Google’s Bigtable, HBase is an open-source, distributed, versioned, column-oriented store that sits on top of HDFS. HBase is column-based rather than row-based, which enables high-speed execution of operations performed over similar values across massive datasets.	http://hbase.apache.org
HCatalog	• An incubator-level project at Apache, HCatalog is a metadata and table storage management service for HDFS.	http://Incubator.apache.org/hcatalog/
Hive	• Hive provides a warehouse structure and SQL-like access for data in HDFS and other Hadoop input sources	http://hive.apache.org
Mahout	• Mahout is a scalable machine-learning and data mining library.	http://mahout.apache.org
Oozie	• Oozie is a job coordinator and workflow manager for jobs executed in Hadoop, which can include non-MapReduce jobs.	http://oozie.apache.org
Pig	• Pig is a framework consisting of a high-level scripting language (Pig Latin) and a run-time environment that allows users to execute MapReduce on a Hadoop cluster.	http://pig.apache.org/docs/r0.7.0/piglatin_ref2.html
Sqoop	• Sqoop (SQL-to-Hadoop) is a tool which transfers data in both directions between relational systems and HDFS or other Hadoop data stores, e.g. Hive or HBase.	http://sqoop.apache.org
ZooKeeper	• ZooKeeper is a service for maintaining configuration information, naming, providing distributed synchronization and providing group services.	http://zookeeper.apache.org
YARN	• YARN is a resource-management platform responsible for managing compute resources in clusters and using them for scheduling of users’ applications.	http://hadoop.apache.org/docs/r2.3.0/hadoop-yarn/hadoop-yarn-site/YARN.html
Cascading	• Cascading is an alternative API to Hadoop MapReduce. Cascading now has support for reading and writing data to and from a HBase cluster.	http://wiki.apache.org/hadoop/Hbase/Cascading
Twitter Storm	• Twitter Storm is a free and open source distributed real time computation system.	http://storm.incubator.apache.org/
High performance computing cluster (HPCC)	• HPCC is an open source, data-intensive computing system platform developed by LexisNexis Risk Solutions	http://hpccsystems.com/
Dremel	• Dremel is a scalable, interactive ad-hoc query system for analysis of read-only nested data	http://research.google.com/pubs/pub36632.html

Relevant literature cited in this paper related to “MapReduce, Hadoop, clinical data, and biomedical/bioinformatics applications of MapReduce” was obtained from PubMed, IEEEXplore, Springer, and BioMed Central databases. The MapReduce programming framework was first introduced to industry in 2006. And thus the literature search concentrated on 2007 to 2014. A total of 32 articles were found based on the use of the MapReduce framework to process the clinical big data and its application using the Hadoop platform.

## Review

In this review we start by listing the different types of big clinical datasets, followed by the efforts that are developed to leverage the data for analytical advantages. These advantages are mainly focused on descriptive and predictive analytics. The major reason for using the MapReduce programming framework in the reviewed efforts is to speed up these kind of analytics. This is due the fact that these kinds of analytic algorithms are very well developed and tested for the MapReduce framework and the Hadoop platform can handle a huge amount of data [11] in a small amount of time. The prescriptive analytics require data sharing among computing nodes, which unfortunately cannot be achieved easily (i.e. sophisticated programs with a great deal of data management) using MapReduce, and thus, not all optimization problems (i.e. prescriptive analytics) can be implemented on the MapReduce framework.

The review section is followed by a challenges and future trends section that highlights the use of the MapReduce programming framework and its open source implementation Hadoop for processing clinical big data. This is followed by our perspective and use cases on how to leverage clinical big data for novel analytics.

### Clinical big data analysis

The exponential production of data in recent years has introduced a new area in the field of information technology known as ‘Big Data’. In a clinical setting such datasets are emerging from large-scale laboratory information system (LIS) data, test utilization data, electronic medical record (EMR), biomedical data, biometrics data, gene expression data, and in other areas. Massive datasets are extremely difficult to analyse and query using traditional mechanisms, especially when the queries themselves are quite complicated. In effect, a MapReduce algorithm maps both the query and the dataset into constituent parts. The mapped components of the query can be processed simultaneously – or reduced – to rapidly return results.

Big datasets of clinical, biomedical, and biometric data have been processed successfully using the MapReduce framework on top of the Hadoop distributed file system. An overview of the Hadoop platform, MapReduce framework and its current applications [26,27] has been reported for the field of bioinformatics. The promise of big data analytics in bioinformatics and health care in general has previously been described [5]. However our review enlarges the scope to the application of the MapReduce framework and its open source implementation Hadoop to a wide range of clinical big data including:

1. Publicly available clinical datasets: online published datasets and reports from the United States Food and Drug Administration (FDA) [28].

2. Biometrics datasets: containing measurable features related to human characteristics. Biometrics data is used as a form of identification and access control [29].

3. Bioinformatics datasets: biological data of a patient (e.g. protein structure, DNA sequence, etc.).

4. Biomedical signal datasets: data resulting from the recording of vital signs of a patient (e.g. electrocardiography (ECG), electroencephalography (EEG), etc.).

5. Biomedical image datasets: data resulting from the scanning of medical images (e.g. ultrasound imaging, magnetic resonance imaging (MRI), histology images, etc.).

Moreover, our review presents a detailed discussion about the various types of clinical big data, challenges and consequences relevant to the application of big data analytics in a health care facility. This review is concluded with the future potential applications of the MapReduce programming framework and the Hadoop platform applied to clinical big data.

### Public databases

A MapReduce-based algorithm [30] has been proposed for common adverse drug event (ADE) detection and has been tested in mining spontaneous ADE reports from the United States FDA. The purpose of this algorithm was to investigate the possibility of using the MapReduce framework to speed up biomedical data mining tasks using this pharmacovigilance case as one specific example. The results demonstrated that the MapReduce programming framework could improve the performance of common signal detection algorithms for pharmacovigilance [30] in a distributed computation environment at approximately linear speedup rates. The MapReduce distributed architecture and high dimensionality compression via Markov boundary feature selection [31] have been used to identify unproven cancer treatments on the World Wide Web. This study showed that unproven treatments used distinct language to market their claims and this language was learnable, and through distributed parallelization and state of the art feature selection [32], it is possible to build and apply models with large scalability.

A novel system known as GroupFilterFormat [33] has been developed to handle the definition of field content based on a Pig Latin script [20]. Dummy discharge summary data for 2.3 million inpatients and medical activity log data for 950 million events were processed. The response time was significantly reduced and a linear relationship was observed between the quantity of data and processing time in both a small and a very large dataset. The results show that doubling the number of nodes resulted in a 47% decrease in processing time.

### Biometrics

The MapReduce programming framework has also been used to classify biometric measurements [34] using the Hadoop platform for face matching, iris recognition, and fingerprint recognition. A biometrics prototype system [35] has been implemented for generalized searching of cloud-scale biometric data and matching a collection of synthetic human iris images. A biometric-capture mobile phone application has been developed for secure access to the cloud [36]. The biometric capture and recognition are performed during a standard Web session. The Hadoop platform is used to establish the connection between a mobile user and the server in the cloud.

### Bioinformatics: genome and protein big data analysis

The large datasets stemming from genomic data are particularly amenable to analysis by distributed systems. A novel and efficient tag for single-nucleotide polymorphism (SNP) selection algorithms has been proposed using the MapReduce framework [37]. A genome sequence comparison algorithm [38] has been implemented on top of Hadoop while relying on HBase [39] for data management and MapReduce jobs for computation. The system performance has been tested with real-life genetic sequences on the level of single genes as well as artificially generated test sequences [38]. While the initial test runs clearly illustrated the feasibility of the approach, more work is needed to improve the applicability of the solution. Moreover additional tuning of the local Hadoop configuration towards the genome comparison is expected to yield additional performance benefits. A bioinformatics processing tool known as BioPig has been built on the Apache’s Hadoop system and the Pig Latin data flow language [40]. Compared with traditional algorithms, BioPig has three major advantages: first, BioPig programmability reduces development time for parallel bioinformatics applications; second, testing BioPig with up to 500 GB sequences demonstrates that it scales automatically with the size of data; and finally, BioPig can be ported without modification on many Hadoop infrastructures, as tested with the Magellan system at the National Energy Research Scientific Computing Center (NERSC [41]) and the Amazon Elastic Compute Cloud [42]. Chang et al. [43] have developed a distributed genome assembler based on string graphs and the MapReduce framework, known as the CloudBrush. The assembler includes a novel edge-adjustment algorithm to detect structural defects by examining the neighbouring areas of a specific read for sequencing errors and adjusting the edges of the string graph. McKenna et al. [44] presented a sequence database search engine that was specifically designed to run efficiently on the Hadoop distributed computing platform. The search engine implemented the K-score algorithm [45], generating comparable output for the same input files as the original implementation for mass spectrometry based proteomics. A parallel protein structure alignment algorithm has also been proposed based on the Hadoop distributed platform [46]. The authors analysed and compared the structure alignments produced by different methods using a dataset randomly selected from the Protein Data Bank (PDB) database [19]. The experimental results verified that the proposed algorithm refined the resulting alignments more accurately than existing algorithms. Meanwhile, the computational performance of the proposed algorithm was proportional to the number of processors used in the cloud platform. The implementation of genome-wide association study (GWAS) statistical tests in the R programming language has been presented in the form of the BlueSNP R package [47], which executes calculations across clusters configured with Hadoop. An efficient algorithm for DNA fragment assembly in the MapReduce framework has been proposed [48]. The experimental results show that the parallel strategy can effectively improve the computational efficiency and remove the memory limitations of the assembly algorithm based on the Euler super path [49]. Next generation genome software mapping has been developed for SNP discovery and genotyping [50]. The software is known as Cloudburst and it is implemented on top of the Hadoop platform for the analysis of next generation sequencing data. Performance comparison studies have been conducted between a message passing interface (MPI) [51], Dryad [52], and a Hadoop MapReduce programming framework for measuring relative performance using three bioinformatics applications [53]. BLAST and gene set enrichment analysis (GSEA) algorithms have been implemented in Hadoop [54] for streaming computation on large data sets and a multi-pass computation on relatively small datasets. The results indicate that the framework could have a wide range of bioinformatics applications while maintaining good computational efficiency, scalability, and ease of maintenance. CloudBLAST [55], a parallelized version of the NCBI BLAST2 algorithm [56] is implemented using Hadoop. The results were compared against the available version of mpiBLAST [57], which is an earlier parallel version of BLAST. CloudBLAST showed better performance and was considered simpler than mpiBLAST. The Hadoop platform has been used for multiple sequence alignment [58] using HBase.

The reciprocal smallest distance (RSD) algorithm for gene sequence comparison has been redesigned to run with EC2 cloud [42]. The redesigned algorithm used ortholog calculations across a wide selection of fully sequenced genomes. They ran over 300,000 RSD process using the MapReduce framework on the EC2 cloud running on 100 high capacity computing nodes. According to their results, MapReduce provides a substantial boost to the process.

Cloudgene [59] is a freely available platform to improve the usability of MapReduce programs in bioinformatics. Cloudgene is used to build a standardized graphical execution environment for currently available and future MapReduce programs, which can be integrated by using its plug-in interface. The results show that MapReduce programs can be integrated into Cloudgene with little effort and without adding any computational overhead to existing programs. Currently, five different bioinformatics programs using MapReduce and two systems are integrated and have been successfully deployed [59].

Hydra is a genome sequence database search engine that is designed to run on top of the Hadoop and MapReduce distributed computing framework [60]. It implements the K-score algorithm [45] and generates comparable output for the same input files as the original implementation. The results show that the software is scalable in its ability to handle a large peptide database.

A parallel version of the random forest algorithm [61] for regression and genetic similarity learning tasks has been developed [62] for large-scale population genetic association studies involving multivariate traits. It is implemented using MapReduce programming framework on top of Hadoop. The algorithm has been applied to a genome-wide association study on Alzheimer disease (AD) in which the quantitative characteristic consists of a high-dimensional neuroimaging phenotype describing longitudinal changes in human brain structure and notable speed-ups in the processing are obtained.

A solution to sequence comparison that can be thoroughly decomposed into multiple rounds of map and reduce operations has been proposed [63]. The procedure described is an effort in decomposition and parallelization of sequence alignment in prediction of a volume of genomic sequence data, which cannot be processed using sequential programming methods.

Nephele is a suite of tools [64] that uses the complete composition vector algorithm [65] to represent each genome sequence in the dataset as a vector derived from its constituent. The method is implemented using the MapReduce framework on top of the Hadoop platform. The method produces results that correlate well with expert-defined clades at a fraction of the computational cost of traditional methods [64]. Nephele was able to generate a neighbor-joined tree of over 10,000 16S samples in less than 2 hours.

A practical framework [66] based on MapReduce programming framework is developed to infer large gene networks, by developing and parallelizing a hybrid genetic algorithm particle swarm optimization (GA-PSO) method [67]. The authors use the open-source software GeneNetWeaver to create the gene profiles. The results show that the parallel method based on the MapReduce framework can be successfully used to gather networks with desired behaviors and the computation time can be reduced.

A method for enhancement of accuracy and efficiency for RNA secondary structure prediction by sequence segmentation and MapReduce has been implemented [68]. The results show that by using statistical analysis implemented using the MapReduce framework, the inversion-based chunking methods can outperform predictions using the whole sequence.

Rainbow [69] is a cloud-based software package that can assist in the automation of large-scale whole-genome sequencing (WGS) data analyses to overcome the limitations of Crossbow [70], which is a software tool that can detect SNPs WGS data from a single subject. The performance of Rainbow was evaluated by analyzing 44 different whole-genome-sequenced subjects. Rainbow has the capacity to process genomic data from more than 500 subjects in two weeks using cloud computing provided by the Amazon Web Service.

Mercury [71] is an automated, flexible, and extensible analysis workflow that provides accurate and reproducible genomic results at scales ranging from individuals to large partners. Moreover, Mercury can be deployed on local clusters and the Amazon Web Services cloud via the DNAnexus platform.

### Biomedical signal analysis

The parallel ensemble empirical mode decomposition (EEMD) algorithm [72] has been implemented on top of the Hadoop platform in a modern cyber infrastructure [73]. The algorithm described a parallel neural signal processing with EEMD using the MapReduce framework. Test results and performance evaluation show that parallel EEMD can significantly improve the performance of neural signal processing. A novel approach has been proposed [39] to store and process clinical signals based on the Apache HBase distributed column-store and the MapReduce programming framework with an integrated Web-based data visualization layer.

### Biomedical image analysis

The growth in the volume of medical images produced on a daily basis in modern hospitals has forced a move away from traditional medical image analysis and indexing approaches towards scalable solutions [74]. MapReduce has been used to speed up and make possible three large–scale medical image processing use–cases: (1) parameter optimization for lung texture classification using support vector machines (SVM), (2) content–based medical image indexing/retrieval, and (3) dimensional directional wavelet analysis for solid texture classification [75]. A cluster of heterogeneous computing nodes was set up using the Hadoop platform allowing for a maximum of 42 concurrent map tasks. The majority of the machines used were desktop computers that are also used for regular office work. The three use–cases reflect the various challenges of processing medical images in different clinical scenarios.

An ultrafast and scalable cone-beam computed tomography (CT) reconstruction algorithm using MapReduce in a cloud-computing environment has been proposed [76]. The algorithm accelerates the Feldcamp-Davis-Kress (FDK) algorithm [77] by porting it to a MapReduce implementation. The map functions were used to filter and back-project subsets of projections, and reduce functions to aggregate that partial back-projection into the whole volume. The speed up of reconstruction time was found to be roughly linear with the number of nodes employed.

Table 3 includes a summary of the discussed literature on clinical big data analysis using the MapReduce programming framework. It tabulates the studies referenced in this paper grouped by relevant categories to indicate the following fields: study name, year, and technology used, and potential application of the algorithm or the technology used.

**Table 3:** Summary of reviewed research in clinical big data analysis using the MapReduce programming model

**Study category**	**Study Name/Reference**	**Study year**	**Technology used**	**Application**
Public database	A drug-adverse event extraction algorithm to support pharmacovigilance knowledge mining from PubMed citations/[30]	2011	A MapReduce based algorithm for common adverse drug events (ADE) detection	Biomedical data mining
	Identifying unproven cancer treatments on the health web: Addressing accuracy, generalizability and scalability/[31]	2012	Using MapReduce and Markove boundary feature selection	Identify unproven cancer treatments on the health web
	A user-friendly tool to transform large scale administrative data into wide table format using a MapReduce program with a pig latin based script/[33]	2012	MapRedcue and Pig Latin	Administrative data management
Biometric	Leveraging the cloud for big data biometrics: Meeting the performance requirements of the next generation biometric systems/[34]	2011	MapReduce machine learning algorithms for image regnition on Hadoop paltform	Design of secuirty system using biometric identification
	Iris recognition on hadoop: A biometrics system implementation on cloud computing/[35]	2011	Human iris MapReduce search algorithm on the cloud	Data retrival and secuirty system
	Cloud-ready biometric system for mobile security access/[36]	2012	MapReduce algorithm to capture and recognition of biometric information	Biometric-identification mobile phone applications
Genome and Protein data analysis	Parallelizing bioinformatics applications with MapReduce/[54]	2008	MapRedcue algorithms	Bioinformatics applications
	Cloudblast: Combining MapReduce and virtualization on distributed resources for bioinformatics applications/[55]	2008	Cloud/MapReduce	Bioinformatics applications
	CloudBurst: highly sensitive read mapping with MapReduce/[50]	2009	MapRedcue algorithms	Genome sequence mapping tool
	Cloud technologies for bioinformatics applications/[53]	2009	Cloud/MapReduce	Bioinformatics applications
	The genome analysis toolkit: A MapReduce framework for analyzing next-generation DNA sequencing data/[44]	2010	HBase for data management and MapReduce jobs for computation	Genome sequence comparison application
	Nephele: genotyping via complete composition vectors and MapReduce/[64]	2011	MapReduce Algorithms	Genotyping sequence tool
	A graphical execution platform for MapReduce programs on private and public clouds/[59]	2012	Cloud/MapReduce	Bioinformatics applications
	Hydra: a scalable proteomic search engine which utilizes the Hadoop distributed computing framework/[60]	2012	MapReduce Algorithms	Bioinformatics applications
	An efficient algorithm for DNA fragment assembly in MapReduce/[48]	2012	MapReduce algorithm for DNA framentation	A tool for DNA fragmentation assembly
	De novo assembly of high-throughput sequencing data with cloud computing and new operations on string graphs/[43]	2012	String graph based on the MapReduce algorithms	Distributed Genome assembler
	Fractal MapReduce decomposition of sequence alignment/[63]	2012	MapReduce Algorithms	Genome sequence alignment tool
	Genotyping in the cloud with crossbow/[70]	2012	Cloud	Genotyping application
	BioPig: A hadoop-based analytic toolkit for large-scale sequence data [40]	2013	MapReduce algorithms	Bioinformatics processing tool known as BioPig
	Implementation of a parallel protein structure alignment service on cloud/[46]	2013	MapReduce alignment algorithm	Protein alignment application
	BlueSNP: R package for highly scalable genome-wide association studies using hadoop clusters/[47]	2013	R alagorithms executed on top of the Hadoop platform	Statistical package in R for Genome analysis
	Enhancement of accuracy and efficiency for RNA secondary structure prediction by sequence segmentation and MapReduce/[68]	2013	MapReduce algorithms	Enhanced algorithm
	Rainbow: a tool for large-scale whole-genome sequencing data analysis using cloud computing/[69]	2013	Cloud	Whole-genome sequencing
Study Category	Study Name/Reference	Study year	Technology used	Application
Genome and Protein data analysis	Random forests on Hadoop for genome-wide association studies of multivariate neuroimaging phenotypes/[62]	2013	MapReduce Algorithms	multivariate neuroimaging phenotypes
	Novel and efficient tag SNPs selection algorithms/[37]	2014	MapReduce algorithm for efficient selection of SNP	Genom analysis
	Designing a parallel evolutionary algorithm for inferring gene networks on the cloud computing environment/[66]	2014	Cloud	Algorithm for inferring gene networks
	Launching genomics into the cloud: deployment of Mercury, a next generation sequence analysis pipeline/[71]	2014	Cloud	sequence analysis application
Biomedical signal analysis	HBase, MapReduce, and integrated data visualization for processing clinical signal data/[39]	2011	HBase for data mangement and MapReduce processing algorithm	Store and processing clinical signals
	Parallel processing of massive EEG data with MapReduce/[73]	2012	MapReduce EEMD algorithm	Massive biomedical signal processing
Biomedical image analysis	Hadoop-gis: A high performance query system for analytical medical imaging with MapReduce/[74]	2011	HBase for data management and MapReduce processing algorithm	Store and processing of medical images
	Ultrafast and scalable cone-beam CT reconstruction using MapReduce in a cloud computing environment [76]	2011	MapReduce image processing algorithms on the Cloud	Accelerates FDK algorithm for the cone-beam CT
	Using MapReduce for Large-Scale Medical Image Analysis/[75]	2012	MapReduce algorithm	Medical Image Analysis

The summary includes information related to the study (i.e. category, name, year, technology used, experiment design and potetial applications).

## Challenges and future trends

### Challenges and consequences

Health care systems in general suffer unsustainable costs and lack data utilization [78]. Therefore there is a pressing need to find solutions that can reduce unnecessary costs. Advances in health quality outcomes and cost control measures depend on using the power of large integrated databases to underline patterns and insights. However, there is much less certainty on how this clinical data should be collected, maintained, disclosed, and used. The problem in health care systems is not the lack of data, it is the lack of information that can be utilized to support critical decision-making [79]. This presents the following challenges to big data solutions in clinical facilities:

1- Technology straggling. Health care is resistant to redesigning processes and approving technology that influences the health care system [80].

2- Data dispersion. Clinical data is generated from many sources (e.g. providers, labs, data vendors, financial, regulations, etc.) this motivates the need for data integration and maintaining mechanism to hold the data into a flexible data warehouse.

3- Security concerns and privacy issues. There are lots of benefits from sharing clinical big data between researchers and scholars, however these benefits are constricted due to the privacy issues and laws that regulate clinical data privacy and access [81].

4- Standards and regulations. Big data solution architectures have to be flexible and adoptable to manage the variety of dispersed sources and the growth of standards and regulations (e.g. new encryption standards that may require system architecture modifications) that are used to interchange and maintain data [82].

### An outlook for the future

Big Data has a substantial potential to unlock the whole health care value chain [83]. Big data analytics changed the traditional perspective of health care systems from finding new drugs to patient-central health care for better clinical outcomes and increased efficiency. The future applications of big data in the health care system have the potential of enhancing and accelerating interactions among clinicians, administrators, lab directors, logistic mangers, and researchers by saving costs, creating better efficiencies based on outcome comparison, reducing risks, and improving personalized care.

The following is a list is of potential future applications associated with clinical big data.

1- E-clinics, E-medicine, and similar case retrieval applications based on text analytics applications.

Large amounts of health data is unstructured as documents, images, clinical or transcribed notes [84]. Research articles, review articles, clinical references, and practice guidelines are rich sources for text analytics applications that aim to discover knowledge by mining these type of text data.

2- Genotyping applications.

Genomic data represent significant amounts of gene sequencing data and applications are required to analysis and understand the sequence in regards to better understanding of patient treatment.

3- Mining and analysis of biosensors applications.

Streamed data home monitoring, tele-health, handheld and sensor-based wireless are well established data sources for clinical data.

4- Social media analytics applications.

Social media will increase the communication between patients, physician and communities. Consequently, analytics are required to analyse this data to underline emerging outbreak of disease, patient satisfaction, and compliance of patient to clinical regulations and treatments.

5- Business and organizational modelling applications.

Administrative data such as billing, scheduling, and other non-health data present an exponentially growing source of data. Analysing and optimizing this kind of data can save large amounts of money and increase the sustainability of a health care facility [78,79,83].

The aforementioned types of clinical data sources provide a rich environment for research and give rise to many future applications that can be analysed for better patient treatment outcomes and a more sustainable health care system.

### Clinical big data and the upcoming challenges

Big data by itself usually confers little direct advantage, however analytics based on big data can reveal many actionable insights that may prove useful in a clinical environment. This section describe the potential benefits and highlight potential application to leverage the clinical big data for analytical advantages using the MapReduce programming framework and the Hadoop platform.

Epilepsy affects nearly 70 Million people around the world [85], and is categorized by the incident of extemporaneous seizures. Many medications can be given at high doses to inhibit seizures [85,86], however patients often suffer side effects. Even after surgical removal of epilepsy foci, many patients suffer extemporaneous seizures [86]. Seizure prediction systems have the potential to help patients alleviate epilepsy episodes [85,86]. Computational algorithms must consistently predict periods of increased probability of seizure incidence. If the seizure states can be predicted and classified using data mining algorithms, implementation of these algorithms on wearable devices can warn patients of impending seizures. Patients could avoid potentially unsuitable activities in potential seizures episode (e.g. driving and swimming). Seizure patterns are wide and complex resulting in a massive datasets when digitally acquired. MapReduce and Hadoop can be consciously used to train detection and forecasting models. Simulation of different concurrently seizures pattern require the development of complex distributed algorithms to deal with the massive datasets.

Understanding how the human brain functions is the main goal in neuroscience research [87,88]. Non-invasive functional neuroimaging techniques, such as magneto encephalography (MEG) [89], can capture huge time series of brain data activities. Analysis of concurrent brain activities can reveal the relation between the pattern of recorded signal and the category of the stimulus and may provide insights about the brain functional foci (e.g. epilepsy, Alzheimer’s disease [90], and other neuro-pathologies, etc.). Among the approaches to analyse the relation between brain activity and stimuli, the one based on predicting the stimulus from the concurrent brain recording is called brain decoding.

The brain contains nearly 100 billion neurons with an average of 7000 synaptic connections each [87,88,91]. Tracing the neuron connections of the brain is therefore a tedious process due to the resulting massive datasets. Traditional neurons visualization methods cannot scale up to very large scale neuron networks. MapReduce framework and Hadoop platform can be used to visualize and recover neural network structures from neural activity patterns.

More than 44.7 million individuals in the United States are admitted to hospitals each year [92]. Studies have concluded that in 2006 well over $30 billion was spent on unnecessary hospital admissions [93]. To achieve the goal of developing novel algorithms that utilize patient data claim to predict and prevent unnecessary hospitalizations. Claims data analytics require text analytics, prediction and estimation models. The models must be tuned to alleviate the potential risk of decline the admission of patients who need to be hospitalized. This type of analysis is one application of fraud analysis in medicine.

## Conclusions

An integrated solution eliminates the need to move data into and out of the storage system while parallelizing the computation, a problem that is becoming more important due to increasing numbers of sensors and resulting data. And, thus, efficient processing of clinical data is a vital step towards multivariate analysis of the data in order to develop a better understanding of a patient clinical status (i.e. descriptive and predictive analysis). This highly demonstrates the significance of using the MapReduce programming model on top of the Hadoop distributed processing platform to process the large volume of clinical data.

Big data solutions [20-24,42] presents an evolution of clinical big data analysis necessitated by the emergence of ultra-large-scale datasets. Recent developments in open source software, that is, the Hadoop project and the associated software projects, provide a backbone foundation for scaling to terabytes and petabytes data warehouses on Linux clusters, providing fault-tolerant parallelized analysis on such data using a programming framework named MapReduce.

The Hadoop platform and the MapReduce programming framework already have a substantial base in the bioinformatics community, especially in the field of next-generation sequencing analysis, and such use is increasing. This is due to the cost-effectiveness of the Hadoop-based analysis on commodity Linux clusters, and in the cloud via data upload to cloud vendors who have implemented Hadoop/HBase; and due to the effectiveness and ease-of-use of the MapReduce method in parallelization of many data analysis algorithms.

HDFS supports multiple reads and one write of the data. The write process can therefore only append data (i.e. it cannot modify existing data within the file). HDFS does not provide an index mechanism, which means that it is best suited to read-only applications that need to scan and read the complete contents of a file (i.e. MapReduce programs). The actual location of the data within an HDFS file is transparent to applications and external software. And, thus, Software built on top of HDFS has little control over data placement or knowledge of data location, which can make it difficult to optimize performance.

Future work on big clinical data analytics should emphasize modelling of whole interacting processes in a clinical setting (e.g. clinical test utilization pattern, test procedures, specimen collection/handling, etc.). This indeed can be constructed using inexpensive clusters of commodity hardware and the appropriate open source tool (e.g. HBase, Hive, and Pig Latin see Table 2 for Hadoop related projects/ecosystems description and definition) to construct convenient processing tools for massive clinical data. These tools will form the basis of future laboratory informatics applications as laboratory data are increasingly integrated and consolidated.

## Competing interests

The authors declare that they have no competing interests.

## Authors’ contributions

EAM, CTN: collection, organizing, and review of the literature; preparing the manuscript. EAM, BHF and CTN: manuscript review, modification, editing, and revision. All authors read and approved the final manuscript.
